# Auditing DNACPR (Do Not Attempt Cardiopulmonary Resuscitation) Documentation Compliance Across Mental Health Inpatient Units in Mersey Care NHS Foundation Trust

**DOI:** 10.1192/bjo.2026.11717

**Published:** 2026-06-30

**Authors:** Sampada Gadde

**Affiliations:** Merseycare NHS Foundation Trust, Liverpool, United Kingdom

## Abstract

**Aims:**

To audit DNACPR (Do Not Attempt Cardiopulmonary Resuscitation) documentation within inpatient units of the Mental Health Care Division at Mersey Care NHS Foundation Trust, assessing compliance with Resus Council and BMA standards.

**Methods:**

This retrospective audit reviewed DNACPR forms identified via the in-house assessment database, with data extracted from electronic systems and case notes. The audit population included 30 patients, of which 8 were applicable during the audit period (January–June 2025). Compliance was assessed against seven defined criteria, including patient information, rationale for DNACPR decision, healthcare professional details, review information, notification, discussion documentation, and CPR alert on RIO.

**Results:**

Overall compliance was **72%**, with an assurance level of **Limited**. High compliance (100%) was achieved for patient information, rationale for DNACPR, healthcare professional details, and CPR alerts on RIO. However, significant gaps were observed in review information (**0% compliance**), notification details (**33% compliance**), and documentation of discussions on RiO (**60% compliance**).

**Conclusion:**

While DNACPR forms generally captured essential patient and professional details, critical areas of non-compliance were identified, particularly around review, notification, and documentation of discussions. These deficiencies pose risks to continuity and quality of care.

